# Primary breast diffuse large B-cell lymphoma mimicking breast carcinoma: A case report and literature review

**DOI:** 10.1097/MD.0000000000050703

**Published:** 2026-09-18

**Authors:** Longquan Li, Hongxi Yao, Siyu Liu, Jieting Yin, Wanqun Li, Aili Saiding

**Affiliations:** a Department of Thyroid and Breast Surgery, Guangyuan Central Hospital Affiliated to North Sichuan Medical College, Guangyuan, China; b Department of Thyroid and Breast Surgery, Guangyuan Central Hospital, Guangyuan, China.

**Keywords:** breast carcinoma, case report, diffuse large B-cell lymphoma, primary breast diffuse large B-cell lymphoma, R-CHOP

## Abstract

**Rationale:**

Primary breast diffuse large B-cell lymphoma (PB-DLBCL) is a rare extranodal manifestation of non-Hodgkin lymphoma (NHL) that can closely resemble breast carcinoma clinically and radiologically. This overlap may delay accurate diagnosis and appropriate treatment. We report a case of PB-DLBCL in a postmenopausal woman whose initial multimodal imaging findings were suspicious for breast carcinoma, but whose final diagnosis was established by core needle biopsy with immunohistochemical and molecular evaluation.

**Patient concerns:**

A 59-year-old postmenopausal woman presented with a painless, palpable mass in the right breast that had been present for approximately 3 weeks.

**Diagnoses:**

Breast ultrasonography, mammography, magnetic resonance imaging (MRI), and positron emission tomography/computed tomography (PET/CT) demonstrated a suspicious right breast mass with ipsilateral axillary lymphadenopathy, initially raising concern for breast carcinoma. Ultrasound-guided core needle biopsy showed diffuse infiltration by atypical lymphoid cells. Immunohistochemistry confirmed a B-cell phenotype, and fluorescence in situ hybridization (FISH) showed no MYC, BCL2, or BCL6 rearrangements. Bone marrow biopsy showed no evidence of marrow involvement. The final diagnosis was PB-DLBCL, non-germinal center B-cell-like (non-GCB) subtype, Ann Arbor stage IIE.

**Interventions:**

The patient received 6 cycles of rituximab, cyclophosphamide, doxorubicin, vincristine, and prednisone (R-CHOP) chemoimmunotherapy. No breast-directed surgery, consolidative radiotherapy, or central nervous system prophylaxis was administered.

**Outcomes:**

At 6 weeks after completion of R-CHOP therapy, PET/CT showed complete metabolic resolution of the right breast lesion, with only a residual punctate calcified focus measuring approximately 0.4 × 0.4 × 0.3 cm. The Deauville score decreased from 5 at baseline to 1 after treatment, meeting Lugano criteria for complete response. No clinically significant treatment-related adverse events were documented.

**Lessons:**

PB-DLBCL can closely mimic breast carcinoma on multimodal imaging. Suspicious breast imaging findings do not exclude lymphoma. Tissue diagnosis with adequate immunophenotypic and molecular evaluation is essential before definitive treatment planning to avoid misdiagnosis and unnecessary surgery.

## 1. Introduction

According to data from the U.S. Surveillance, Epidemiology, and End Results Program, the annual incidence of non-Hodgkin lymphoma is approximately 18.7 cases per 100,000 persons, with the highest prevalence among adults aged 65–74 years.^[1]^ Primary breast lymphoma is a rare extranodal presentation of NHL, accounting for <0.5% of all malignant breast tumors and approximately 0.38–0.7% of all NHL cases.^[2,3]^ Diffuse large B-cell lymphoma (DLBCL) is the most common histologic subtype.^[4]^

Clinically, primary breast lymphoma most often presents as a painless, unilateral breast mass, and its physical examination and imaging findings frequently resemble those of breast carcinoma.^[5]^ Diagnosis of PB-DLBCL depends on histopathologic confirmation. Core needle biopsy with immunohistochemistry is essential for establishing the diagnosis and determining the immunophenotypic subtype. FISH should be considered when high-grade B-cell lymphoma with MYC and BCL2 and/or BCL6 rearrangements is suspected, because these findings carry prognostic and therapeutic implications.^[6]^ Because PB-DLBCL often presents with nonspecific imaging findings that resemble breast carcinoma, timely biopsy is critical to avoid misdiagnosis and inappropriate breast surgery. Treatment is primarily based on systemic chemoimmunotherapy, whereas surgery is generally limited to diagnostic tissue acquisition or the management of selected complications.^[7–10]^

This report describes a case of PB-DLBCL that was initially suspected to be breast carcinoma on imaging. The case highlights the diagnostic challenge of this entity and emphasizes the importance of core needle biopsy, immunophenotypic analysis, molecular testing, and multidisciplinary review in avoiding misdiagnosis and guiding treatment. This case report was prepared in accordance with the CARE case report guideline. All procedures were conducted in accordance with the ethical principles of the Declaration of Helsinki. In accordance with institutional policy, institutional review board approval was not required for this single-patient case report. Written informed consent for publication of deidentified clinical information and accompanying images was obtained from the patient.

## 2. Case presentation

### 2.1. Initial presentation

A 59-year-old postmenopausal woman first noticed a painless, palpable lump in the right breast approximately 3 weeks before presentation. Initial ultrasonography at an outside facility identified a periareolar right breast lesion measuring approximately 3.0 × 1.5 cm, with features concerning for infiltrative growth and possible internal necrosis. She was referred to our breast surgery department for further evaluation. She had no fever, night sweats, unintentional weight loss, or other constitutional symptoms. She denied breast pain, nipple discharge, nipple retraction, pruritus, or skin changes. Her medical history was unremarkable, with no prior breast disease, breast trauma, or major chronic illness. She reported no family history of breast carcinoma or of known hereditary cancer syndrome. On examination, a firm, ill-defined, poorly mobile mass measuring approximately 3.0 × 2.0 cm was palpated in the upper-outer quadrant of the right breast at the 11-o’clock position. There was no tenderness, skin edema or peau d’orange, skin dimpling, nipple inversion, nipple discharge, or nipple deformity. No palpable cervical, supraclavicular, or axillary lymphadenopathy was detected. Routine laboratory tests and serum tumor markers, including CA 15–3, CA125, and carcinoembryonic antigen, were within institutional reference ranges.

### 2.2. Imaging findings

**Figure 1.**
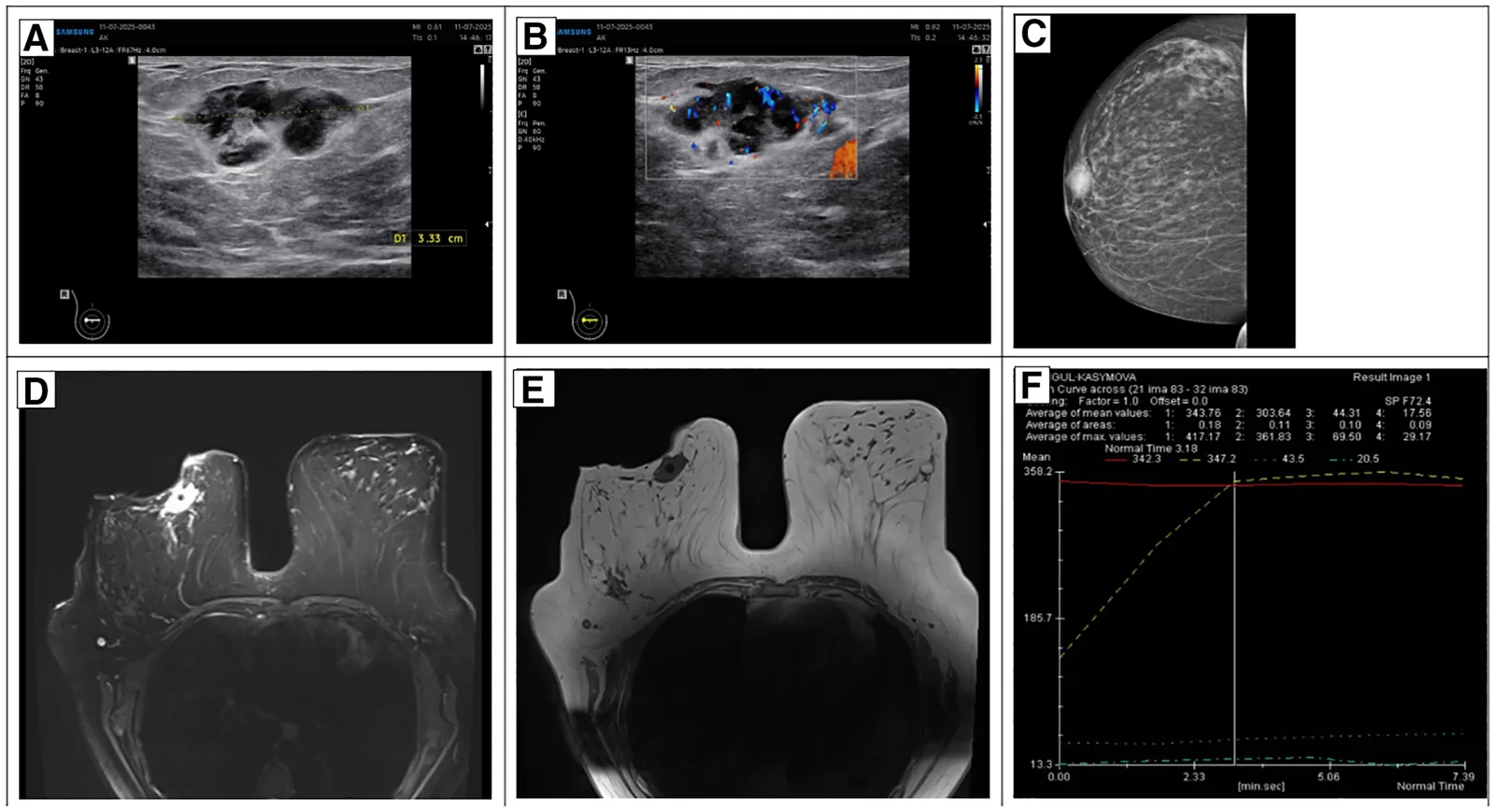
Imaging findings of primary breast diffuse large B-cell lymphoma in this patient. (A) Breast ultrasonography showed a hypoechoic mass with an ill-defined margin, an irregular shape, and heterogeneous internal echogenicity. (B) Color Doppler Flow Imaging demonstrated peripheral vascular signals around the lesion. (C) Mammography showed a high-density lesion with an ill-defined margin. (D, E) Breast magnetic resonance imaging demonstrated a lobulated mass measuring 24 × 29 mm in the upper-outer quadrant of the right breast. The lesion showed slightly prolonged T1- and T2-weighted signal intensity. (F) The time–signal intensity curve showed a plateau pattern, suggesting a possible malignant lesion.

Breast ultrasonography showed a hypoechoic mass measuring 33.3 × 30.8 × 15.7 mm at the 11-o’clock position in the right breast. The mass was irregular, poorly circumscribed, and heterogeneous in echotexture (Fig. 1A). Color Doppler imaging demonstrated increased peripheral vascularity (Fig. 1B). The lesion was classified as Breast Imaging Reporting and Data System (BI-RADS) category 4B. Mammography demonstrated an irregular, high-density mass measuring 30 × 29 mm with indistinct margins in the upper-outer quadrant of the right breast (Fig. 1C), corresponding to BI-RADS category 4A. Breast MRI demonstrated a lobulated mass measuring 24 × 29 mm in the upper-outer quadrant of the right breast (Fig. 1D and E). The lesion showed mildly increased signal intensity on T1- and T2-weighted images, restricted diffusion on diffusion-weighted imaging, and low apparent diffusion coefficient values. Contrast-enhanced MRI showed heterogeneous rim enhancement, and the time-signal intensity curve demonstrated a type II plateau pattern (Fig. 1F). The MRI findings were classified as BI-RADS category 4C. Although serum breast tumor markers were within reference ranges, the overall imaging assessment indicated a suspicious malignant breast lesion. After the risks and benefits were discussed with the patient, an ultrasound-guided core needle biopsy was performed.

### 2.3. Pathologic findings

Ultrasound-guided core needle biopsy of the right breast mass revealed diffuse infiltration of the breast tissue by atypical lymphoid cells. Immunohistochemical staining demonstrated a B-cell phenotype: the tumor cells were positive for CD20, CD79a, CD19, and CD22 and were negative for epithelial markers, including AE1/AE3, E-cadherin, and CK5/6. These findings argued against primary breast carcinoma. According to the Hans algorithm, the tumor was classified as the non-GCB subtype because it was CD10-negative and BCL6-negative with MUM1 positivity. The tumor cells expressed BCL2, and approximately 20% of tumor cells expressed MYC. The Ki-67 proliferation index was approximately 80%. FISH analysis detected no MYC, BCL2, or BCL6 rearrangements, thereby excluding high-grade B-cell lymphoma with MYC and BCL2 and/or BCL6 rearrangements.

### 2.4. Further diagnostic workup and staging

**Figure 2.**
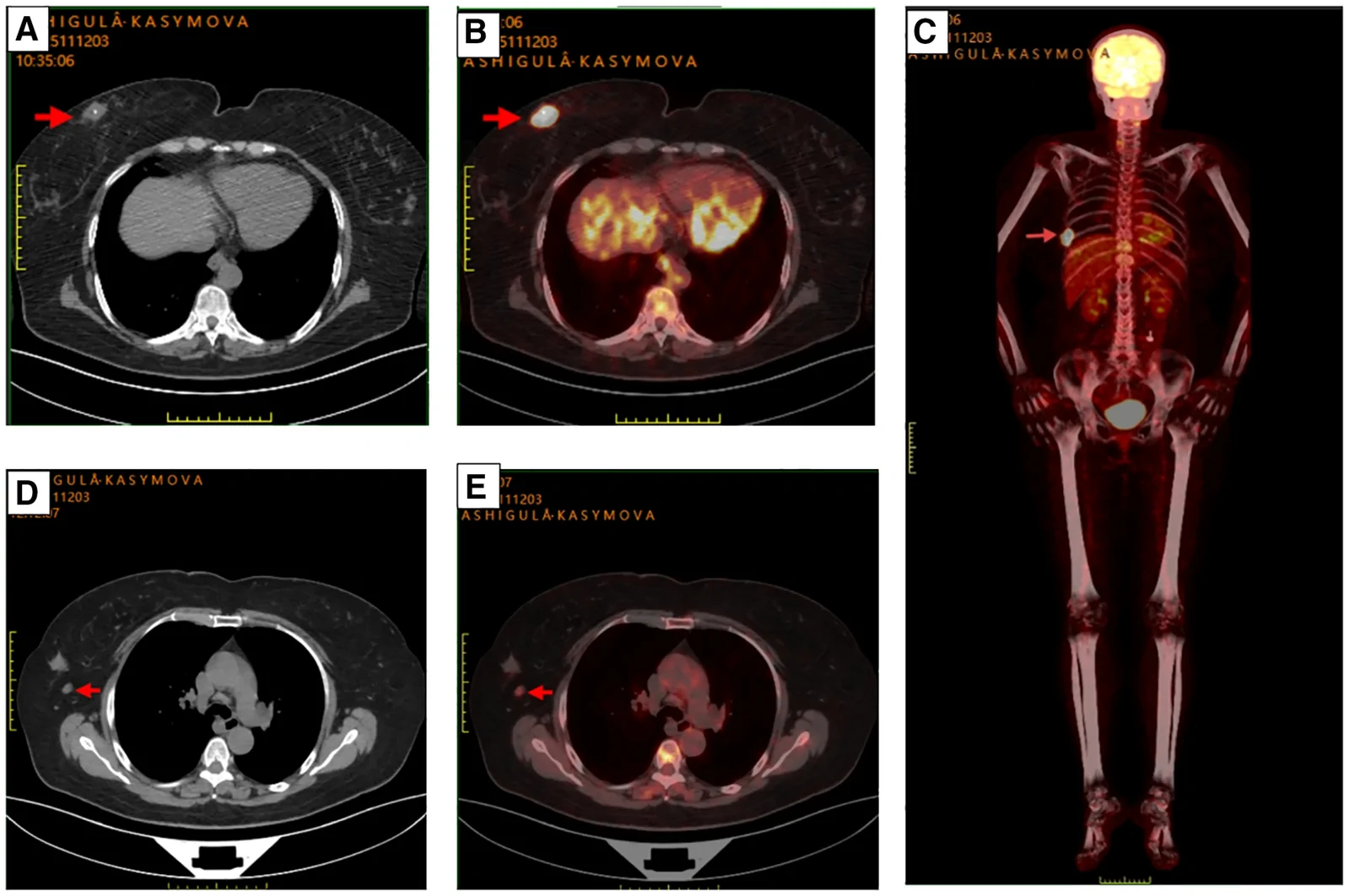
Positron emission tomography/computed tomography (PET/CT) findings. (A) PET/CT showed an isodense solid nodule with punctate calcifications in the right breast (arrow). (B, C) Markedly increased ^18F-fluorodeoxyglucose (^18F-FDG) uptake was observed in the lesion (arrows). (D) Enlarged lymph nodes in the right axilla (1.1 × 1.0 cm, arrow). (E) Enlarged right axillary lymph nodes showed mildly increased metabolic activity (arrow). PET/CT = positron emission tomography/computed tomography; ^18F-FDG = ^18F-fluorodeoxyglucose

Because PB-DLBCL is rare and its management differs from that of breast carcinoma, this case was reviewed at a multidisciplinary team (MDT) conference, and systemic staging was recommended. PET/CT demonstrated multiple isodense nodules in the upper-outer quadrant of the right breast, the largest measuring 3.0 × 1.6 cm, with irregular margins and punctate calcifications (Fig. 2A). The lesion showed markedly increased ^18F-fluorodeoxyglucose (^18F-FDG) uptake, with a maximum standardized uptake value (SUVmax) of 28.4 on early-phase imaging and 47.0 on delayed-phase imaging (Fig. 2B and C). Multiple enlarged right axillary lymph nodes were identified, measuring up to 1.1 × 1.0 cm, with mildly increased metabolic activity (early-phase SUVmax, 1.4; delayed-phase SUVmax, 2.9) (Fig. 2D and E). Bone marrow biopsy showed no abnormalities and no evidence of marrow involvement. The case met the Wiseman and Liao criteria for primary breast lymphoma: adequate pathologic material was available for diagnosis, lymphoma infiltration was anatomically associated with breast tissue, the patient had no previous history of extramammary lymphoma, and systemic staging showed no disseminated disease. Ipsilateral axillary lymph node involvement did not preclude the diagnosis. Because the disease involved the right breast and ipsilateral axillary lymph nodes without distant extranodal disease or bone marrow involvement, the disease was classified as Ann Arbor stage IIE.

### 2.5. Differential diagnosis

The leading differential diagnosis was invasive breast carcinoma, given the patient’s age, unilateral firm breast mass, and suspicious findings on ultrasonography, mammography, and MRI.^[11]^ However, the absence of epithelial marker expression and the presence of B-cell marker expression excluded primary breast carcinoma and supported PB-DLBCL. Other lymphoproliferative disorders, including other small B-cell lymphomas and secondary breast involvement by systemic lymphoma, were considered less likely based on morphologic, immunophenotypic, PET/CT, and bone marrow biopsy findings.

### 2.6. Treatment

After MDT review, the patient was diagnosed with right-sided PB-DLBCL, non-GCB subtype, Ann Arbor stage IIE. Given the localized extranodal disease, ipsilateral nodal involvement, tumor burden, and institutional treatment approach, she received 6 cycles of R-CHOP chemoimmunotherapy. No breast-directed surgery, consolidative radiotherapy, or central nervous system prophylaxis was administered.

### 2.7. Outcome and follow-up

**Figure 3.**
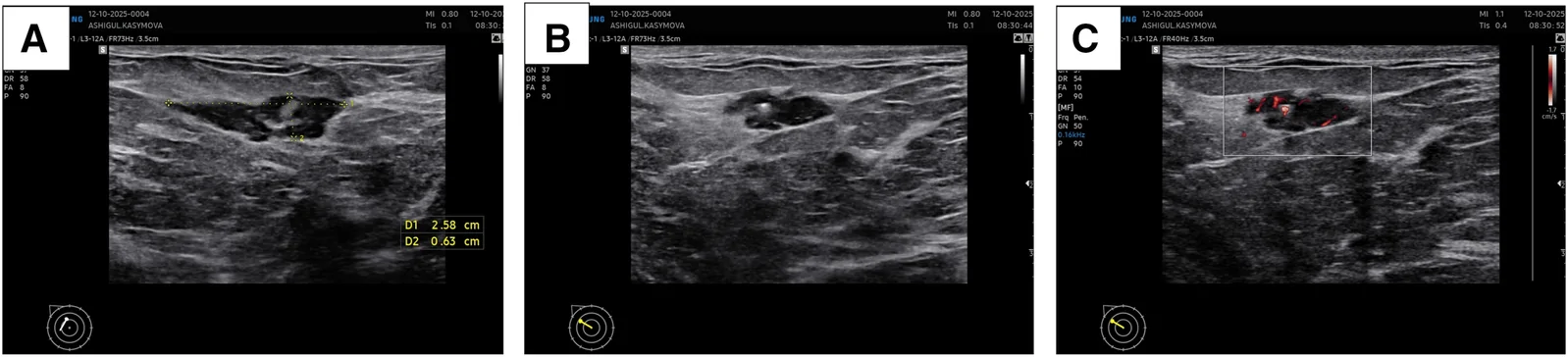
Follow-up ultrasonographic imaging findings after completion of the first treatment cycle. (A) Ultrasonography showed a hypoechoic mass measuring approximately 25.8 × 6.3 × 14 mm at the 11-o’clock position of the right breast. (B) The mass had relatively well-defined margins and an irregular shape, with internal septation-like echoes and punctate hyperechoic foci. (C) Color Doppler flow imaging demonstrated linear blood flow signals within the lesion.

**Figure 4.**
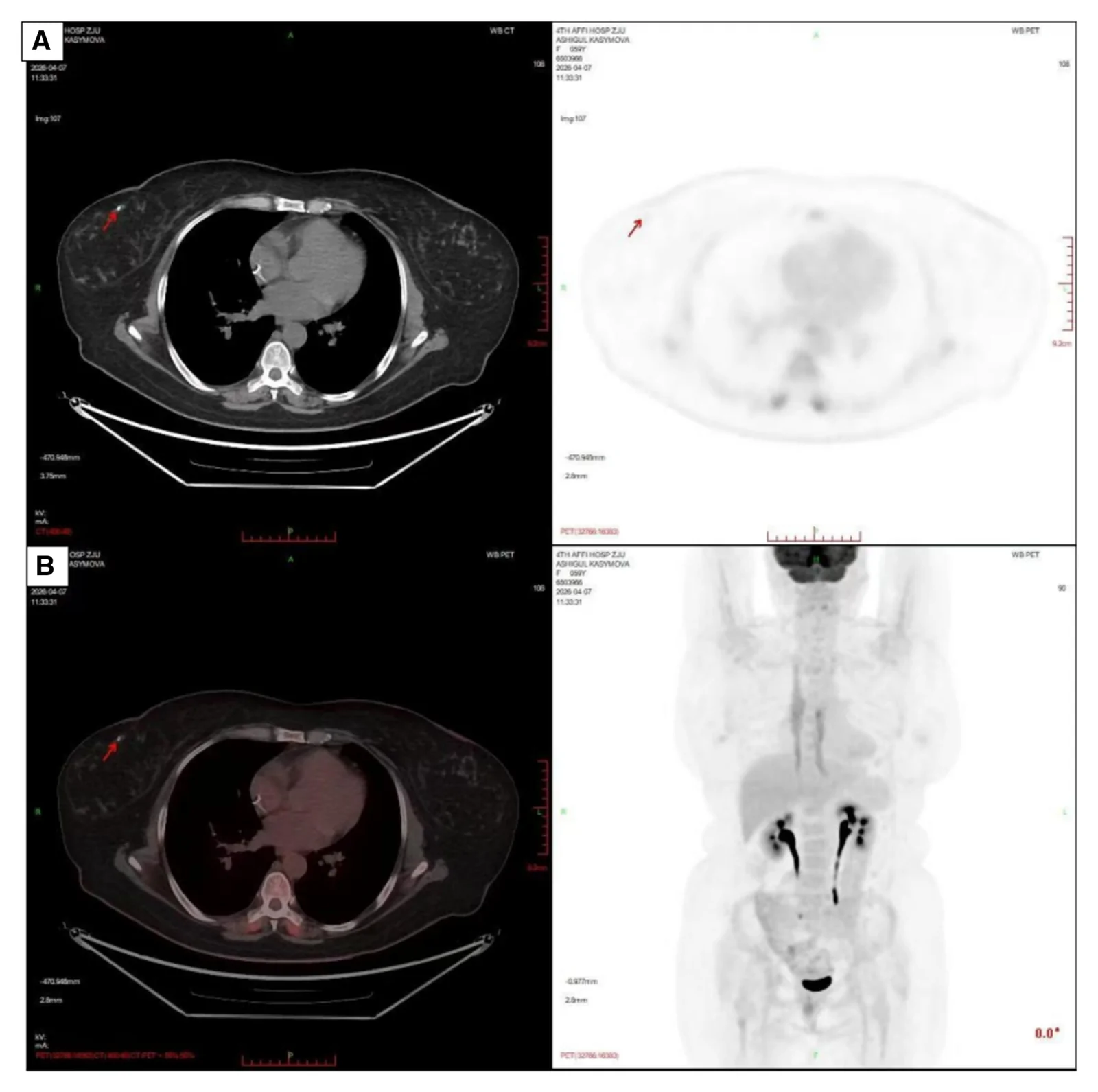
posttreatment follow-up PET/CT images of the patient. (A) PET/CT showed a punctate calcific density measuring approximately 0.4 × 0.4 × 0.3 cm in the upper-outer quadrant of the right breast (arrows). (B) No abnormal ^18F-FDG uptake (arrow).

After the first treatment cycle, follow-up breast ultrasonography showed a 22.5% reduction in maximum tumor diameter (Fig. 3A–C), indicating an early treatment response. Six weeks after completion of 6 cycles of R-CHOP, follow-up PET/CT showed only a punctate calcified focus measuring approximately 0.4 × 0.4 × 0.3 cm in the upper-outer quadrant of the right breast (Fig. 4A), without abnormal ^18F-FDG uptake (Fig. 4B). The posttreatment Deauville score was 1, consistent with complete metabolic response according to the Lugano response criteria. Lactate dehydrogenase remained within the institutional reference range. These findings indicated a complete response to therapy. At the most recent follow-up, the patient remained clinically asymptomatic, with no evidence of relapse. No additional treatment was planned. Because the follow-up interval remains relatively short, continued surveillance is required to assess the durability of remission. The patient reported relief from physical symptoms and treatment-related psychological distress.

## 3. Discussion

PB-DLBCL is an uncommon extranodal lymphoma with substantial clinical and imaging overlap with breast carcinoma.^[11]^ Similar to previously reported cases,^[12,13]^ the present patient was a postmenopausal woman who presented with a painless unilateral breast mass and no constitutional symptoms. Prior reports have described ultrasonographic, mammographic, and MRI findings that may appear suspicious for breast carcinoma; however, these features are not sufficiently sensitive or specific to establish a definitive diagnosis (Table 1).^[14,15]^ In this case, multimodal breast imaging yielded BI-RADS category 4 assessments across modalities, supporting a high suspicion for malignancy. Nevertheless, several features were not specific for breast carcinoma, including the absence of abnormal serum breast tumor markers and the eventual demonstration of lymphoid rather than epithelial differentiation on biopsy. This report adds to the limited literature showing that PB-DLBCL should remain in the differential diagnosis for a suspicious breast mass, particularly when imaging findings are malignant-appearing but not pathognomonic for carcinoma.

**Table 1:** Main clinical manifestations and imaging features of primary breast diffuse large B-cell lymphoma.

Study	Category	Main Findings
Cheah^[14]^ 2024	Clinical manifestations	The median age at diagnosis is approximately 62–64 years in Western countries and 45–53 years in East Asian countries. The disease occurs predominantly in women. Most patients present with a painless breast mass, with relatively frequent involvement of the right breast. Systemic symptoms are uncommon, whereas skin changes, nipple retraction, and nipple discharge are rare.
Surov^[15]^ 2024	Ultrasonographic findings	Lesions commonly appear as homogeneous hypoechoic masses with an oval or round shape and well-defined or slightly lobulated margins. Some lesions show abundant vascularity.
	Mammographic findings	Most lesions appear as round or oval masses with well-defined or slightly lobulated margins. Calcification is uncommon.
	MRI findings	Some patients may present with hyperintense or enhancing masses. On T2-weighted images, lesions are usually hyperintense, whereas on T1-weighted images, they are mostly isointense. The time–signal intensity curve may show a type II plateau pattern.
	CT findings	Most lesions are round or oval and may demonstrate moderate to marked enhancement.

CT = computed tomography, MRI = magnetic resonance imaging.

Histopathologic confirmation remains the cornerstone of the diagnosis of PB-DLBCL. Without timely tissue diagnosis, PB-DLBCL may be misclassified as breast carcinoma, potentially leading to unnecessary mastectomy, axillary surgery, or other breast-directed procedures that do not address the systemic nature of lymphoma. For suspicious breast masses, ultrasound-guided core needle biopsy is an appropriate initial diagnostic approach when the lesion is sonographically visible. It provides tissue for morphologic evaluation, immunophenotyping, and molecular studies while minimizing the risk of unnecessary excisional biopsy or definitive breast surgery. According to the fifth edition of the World Health Organization Classification of Hematolymphoid Tumors, DLBCL can be further characterized by cell of origin as germinal center B-cell-like, activated B-cell-like, or unclassified/type 3 DLBCL.^[16]^ In clinical practice, the Hans algorithm is commonly used as an immunohistochemical surrogate for cell-of-origin classification, categorizing DLBCL into GCB and non-GCB subtypes. According to the Hans algorithm, the GCB subtype is defined by CD10 positivity or by a CD10-negative, BCL6-positive, MUM1-negative phenotype. Other patterns are generally categorized as non-GCB. In the present case, core needle biopsy showed diffuse infiltration of breast tissue by atypical lymphoid cells. Immunohistochemistry confirmed the B-cell lineage, whereas epithelial markers were negative. These findings established DLBCL and excluded primary breast carcinoma. According to the Hans algorithm, this tumor was classified as non-GCB. This distinction is clinically relevant because non-GCB DLBCL is generally associated with more aggressive biology and less favorable outcomes than GCB DLBCL in many series, although prognosis in PB-DLBCL also depends on stage, age, performance status, treatment response, and molecular features.^[17,18]^

CD10 and BCL6 expression typically supports germinal center B-cell differentiation, whereas MUM1 positivity supports post-germinal center differentiation. Co-expression of MYC and BCL2 proteins, when present above established thresholds, has been associated with inferior outcomes in DLBCL.^[17,19,20]^ In this case, the tumor expressed BCL2, approximately 20% of tumor cells expressed MYC, and the Ki-67 proliferation index was approximately 80%, indicating high proliferative activity. FISH detected no MYC, BCL2, or BCL6 rearrangements, thereby excluding high-grade B-cell lymphoma with MYC and BCL2 and/or BCL6 rearrangements.

This case underscores the importance of integrating morphology, immunophenotyping, molecular testing, and systemic staging to establish an accurate diagnosis, assign an appropriate classification, and individualize treatment. For suspected PB-DLBCL, confirming the breast as the primary site and completing accurate staging are essential. PET/CT and bone marrow biopsy are important for excluding disseminated systemic lymphoma, assessing nodal and extranodal disease burden, and completing Ann Arbor staging.^[21]^ In this patient, PET/CT was also useful for response assessment. After treatment, PET/CT demonstrated a complete metabolic response, with the Deauville score decreasing from 5 to 1, meeting Lugano criteria for complete response.

From a therapeutic perspective, PB-DLBCL differs fundamentally from primary breast carcinoma. Systemic rituximab-based chemoimmunotherapy, most commonly R-CHOP, is the mainstay of treatment.^[9,22,23]^ Surgery has no established therapeutic role beyond obtaining diagnostic tissue or managing selected complications.^[9,10]^ The role of consolidative radiotherapy remains individualized and may depend on stage, response, tumor bulk, and institutional practice.^[9,22–24]^ Similarly, central nervous system prophylaxis remains controversial and is generally considered on the basis of patient-specific risk factors rather than breast involvement alone.^[23,25]^

This report has several limitations. As a single-patient case report, it cannot define the optimal management strategy for PB-DLBCL, including the roles of consolidative radiotherapy and central nervous system prophylaxis, both of which remain debated in the literature. In addition, the follow-up interval remains relatively short, precluding conclusions about long-term disease control, relapse patterns, or survival. Nevertheless, this case is clinically instructive because it illustrates a practical diagnostic pitfall in breast practice and emphasizes the importance of biopsy-based diagnosis before treatment selection.

## 4. Conclusions

PB-DLBCL is a rare but important mimic of breast cancer. Because its clinical and radiologic features may strongly suggest breast cancer, definitive diagnosis depends on core needle biopsy, immunohistochemistry, and, when indicated, molecular testing. Early recognition of this entity can prevent unnecessary surgery and facilitate timely initiation of effective systemic therapy.

## Author contributions

**Data curation:** Longquan Li, Hongxi Yao, Siyu Liu, Jieting Yin, Wanqun Li.

**Investigation:** Longquan Li, Hongxi Yao, Aili Saiding.

**Methodology:** Longquan Li, Hongxi Yao, Jieting Yin, Wanqun Li, Aili Saiding.

**Visualization:** Longquan Li, Siyu Liu.

**Writing—original draft:** Longquan Li, Hongxi Yao, Jieting Yin, Wanqun Li.

**Writing—review & editing:** Longquan Li, Aili Saiding.

**Resources:** Siyu Liu.

**Conceptualization:** Aili Saiding.

**Funding acquisition:** Aili Saiding.
